# Fiscal Challenges and Anticipated Changes to Kentucky’s Population Health System

**DOI:** 10.13023/jah.0203.01

**Published:** 2020-07-19

**Authors:** Jeffrey Howard

**Affiliations:** Resident Physician, University of Louisville, Department of Surgery Adjunct Assistant Professor of Public Health, University of Louisville, School of Public Health and Information Science, Former Kentucky Commissioner for Public Health (2017–2019)

**Keywords:** Appalachia, public health, organization of care, rural health, population health, delivery of care, geographic variation

## Abstract

The hallmark of public health is population-level intervention. However, current public health funding in Kentucky is largely programmatic or disease-based. As a result, public health leaders are not able to appropriately utilize present resources to pursue population health endeavors. However, a recent transformation of the public health system has emphasized multisector partnerships and efficient funding mechanisms that may increase resources to pursue population-level health interventions based on community health assessments.

Marked disparities in health outcomes in rural versus urban populations have been well established and permeate nearly all manner of disease. Understanding the antecedent causes of these disparities is challenging due to their complex and multifactorial nature. Notwithstanding the required effort, it is necessary to demonstrate the structural inadequacies of existing population health systems to inform policy and guide public health intervention. To that end, the authors of the article in the current issue of the *Journal of Appalachian Health*^1^ examine population-level health protections and organization of population health systems in urban, rural, and rural Appalachian communities across Kentucky. As hypothesized, the authors found rural Appalachian communities to have limited capacity to deliver population health services.

Examination of public health funding provides context for the authors’ findings. In Kentucky, public health funding combines federal, state, and local sources with the federal portion accounting for more than 50% of total funding. Federal resources generally flow through the state Department for Public Health (DPH) to the local health departments (LHDs), which act as the effector arms of the system. Unfortunately, this funding portion is largely programmatic and often tied to a specific disease. State funding is comparatively minor and largely expended on salaries, including corresponding pension debt, and fills deficits in existing programs. As such, officials at the state and local level do not have the flexibility, except for the use of clever programmatic design, to distribute funding to identified gaps, such as the 20 population health interventions utilized by the authors. A significant portion of local funding is revenue from direct clinical services, which often struggle to generate break-even and thus siphon resources from potential population health interventions. Although population health intervention is the hallmark of public health, current resource allocation has not followed a parallel design and does not afford public health leaders the necessary flexibility to identify and implement community-specific population-level interventions.

The authors^1^ utilize cross-sectional data from the 2018 National Longitudinal Survey of Public Health Systems (NALSYS). Of relevance, in the same year, the Kentucky General Assembly passed pension reform intended to address the state’s pension debt. As a result, added pension costs threatened the fiscal solvency of nearly one-half of the state’s LHDs, many of which are rural. This circumstance compelled state and local leaders to pursue a transformation of the entire public health system. This endeavor culminated in the passage of House Bill 129 during the 2020 legislative session, which codified policies outlined in the final transformation plan. Briefly summarized, the project identified Core public health services, which include statutorily defined programs: the Women, Infants, and Children program; the Health Access Nurturing Development Services program; and harm-reduction programs. These programs, identified because of their importance to the health outcomes of the state, are given priority status for available funding. Programs beyond the Core offering are identified by LHDs on recurrent community health assessments and ranked ordered by priority for available funding. A key component of the transformation is the cultivation of multisector partners to ensure efficient delivery of services and maximize resources for priorities identified by community health assessments. Future studies should evaluate the results of NALSYS surveys to identify changes in population health interventions, the development of multisector partnerships, and changes to the organization of public health entities.

Research, such as that described in this article,^1^ is necessary to inform policy development, which is the ultimate tool of public health. There have been positive indicators that rural issues are gaining the attention of policymakers. For example, in 2018, the Centers for Medicare & Medicaid Services (CMS) published the Centers’ first-ever Rural Health Strategy, which includes the aim of evaluating CMS policy through a rural lens,^2^ and the Administrator has alluded to future rurally-oriented policies.^3^ To address rural and Appalachian population-level health disparities in Kentucky, greater flexibility in existing funding is necessary, and the General Assembly should substantially increase the state allocation to DPH and thereby LHDs. These changes will allow public health leaders to assess population health needs, identify gaps, inform policy, and design interventions best suited to drive positive outcomes.
